# Multimodal treatment of chronic thromboembolic pulmonary hypertension: initial experience at a university hospital in southern Brazil

**DOI:** 10.36416/1806-3756/e20240231

**Published:** 2024-12-17

**Authors:** William Lorenzi, Rodrigo Vugman Wainstein, Roger Pirath Rodrigues, Igor Gorski Benedetto, Marcelo Basso Gazzana

**Affiliations:** 1. Serviço de Cirurgia Torácica, Hospital de Clínicas de Porto Alegre, Universidade Federal do Rio Grande do Sul, Porto Alegre (RS) Brasil.; 2. Serviço de Cardiologia, Hospital de Clínicas de Porto Alegre, Universidade Federal do Rio Grande do Sul, Porto Alegre (RS) Brasil.; 3. Serviço de Pneumologia, Hospital Universitário Professor Polydoro Ernani de São Thiago, Universidade Federal de Santa Catarina, Florianópolis (SC) Brasil.; 4. Programa de Pós-Graduação em Pneumologia, Universidade Federal do Rio Grande do Sul, Porto Alegre (RS) Brasil.; 5. Serviço de Pneumologia, Hospital de Clínicas de Porto Alegre, Universidade Federal do Rio Grande do Sul, Porto Alegre (RS) Brasil.

## TO THE EDITOR:

Chronic thromboembolic pulmonary hypertension (CTEPH) is a rare disease. The current classification of pulmonary hypertension divides it into five main groups based on the underlying cause, with CTEPH being the main cause in group 4.^1^ Nonresolution of pulmonary arterial thrombi is the underlying pathophysiological mechanism. When associated with hyperflow vasculopathy in patent vessels, the disease progresses, culminating in right heart failure and death. The preferred treatment is pulmonary endarterectomy (PEA), which is indicated for patients with resectable lesions and acceptable surgical risk. For inoperable cases, drug treatment and balloon pulmonary angioplasty (BPA) are available. Here, we report two cases of patients treated with the currently recommended multimodal approach. 

**Figure 1:**
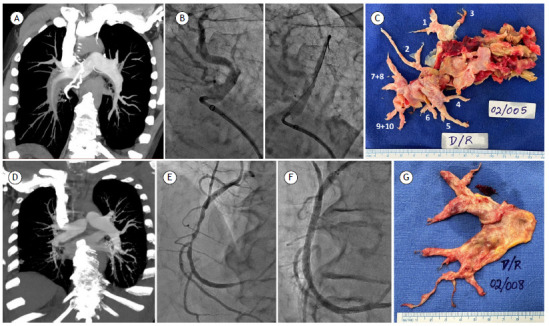
Multimodal treatment of two patients (patient 1, Figures 1A-D; patient 2, Figures 1E-H) with chronic thromboembolic pulmonary hypertension. In A, CT angiography of the chest showing the anatomical difference between the right and left sides. In B, stenotic lesion, left A3 branch. In C, balloon pulmonary angioplasty of the same branch with a 3 mm contrast-filled balloon and a guidewire. In D, surgical specimen, right side. The numbers indicate the corresponding arterial branches. In E, CT angiography of the chest showing central lesions on the right and peripheral lesions on the left. In F, right coronary artery showing multiple stenotic lesions. In G, right coronary artery showing clear flow improvement after stenting. In H, surgical specimen, right side.

The first patient was a 59-year-old nonsmoker male who had been diagnosed with CTEPH three years before. Warfarin, furosemide, and a phosphodiesterase-5 inhibitor were prescribed. In March of 2023, the patient was evaluated by the pulmonary circulation team at the *Hospital de Clínicas de Porto Alegre*, located in the city of Porto Alegre, Brazil. His WHO functional class was IV, and he presented with clinical signs of right heart failure. A transthoracic echocardiogram showed a dilated right ventricle, as well as severe systolic dysfunction and elevated pulmonary artery systolic pressure (Table 1). Perfusion scintigraphy, CT angiography of the chest, and pulmonary arteriography showed complete occlusion of branches to the right middle and lower lobes (Figure 1), as well as bilateral stenotic, web-like lesions in branches to the other lung lobes. Right heart catheterization confirmed severe precapillary pulmonary hypertension (Table 1), with an estimated 1-year mortality > 20%.^1^ During our multidisciplinary board meeting, the disease was considered surgically accessible, despite a very high surgical risk. The left lung lesions were treatable by PEA and BPA. A multimodal approach was proposed, including switching from tadalafil to riociguat; BPA on the left side; and PEA on the right. Between April and July of 2023, the patient underwent four BPA sessions aimed at reducing pulmonary vascular resistance and improving rehabilitation, thus reducing the surgical risk. Three to six branches were treated in each session (Figures 1). Selective segmental and subsegmental angiographies were performed with 6 Fr JR4, multipurpose, and JL3.5 guide catheters. The lesions in branches A1, A3, A5, A6, A7, A8, A9, and A10 were crossed with HI-TORQUE™ balance middleweight universal and Runthrough™ guidewires (Abbott Cardiovascular, Plymouth, MN, USA; and Terumo Corporation, Tokyo, Japan, respectively) and dilated with Euphora™ (Medtronic, Minneapolis, MN, USA) ballon catheters of 2.0 mm × 20 mm, 2.5 mm × 20 mm, 3.0 mm × 20 mm, 3.5 mm × 20 mm, and 4.0 mm × 20 mm for improvement in antegrade flow and venous return. There were no immediate complications. In September of 2023, PEA was performed under extracorporeal circulation (ECC), cooling down to 19°C. PEA was performed on the right side only, with circulatory arrests of 20 min and 12 min. The left side was not explored. Total ECC time was 240 min, and aortic cross-clamp time was 68 min. Anticoagulation with unfractionated heparin began 6 h after surgery. The patient was extubated on postoperative day (POD) 1. Pacemaker wires and drains were removed between PODs 2 and 4, and the patient was discharged from the ICU on POD 4. He was started on warfarin and was sent home on POD 10. Riociguat was discontinued. At two months after surgery, the patient was in WHO functional class I, showing better hemodynamics and exercise capacity (Table 1). 

**Table 1:** Clinical data before and after multimodal treatment of chronic pulmonary hypertension.

	Patient 1			Patient 2		
	Baseline on tadalafil	Post-riociguat + BPA	Post-surgery	Baseline on sildenafil	Post-riociguat + PCI + BPA	Post-surgery
WHO FC	IV	II	I	III	I	I
6MWD, m	211	350	475	200	353	378
BNP, pg/mL	526	221	54	408	210	35
TRV, m/s	4.2	3.6	2.3	4.3	4.2	2.7
PASP, mmHg	84	67	30	90	76	34
TAPSE, mm	14	15	18	15	12	8
RVFAC, %	15	25	33	24	18	30
mPAP, mmHg	47	38	22	51	45	21
CO, L/min	3.4	4.3	5.4	6.1	5.2	6.4
TPR, WU m^2^	13.8	8.8	4.1	8.3	8.6	3.2
RAP, mmHg	8	10	6	8	9	5

BPA: balloon pulmonary angioplasty; PCI: percutaneous coronary intervention; FC: functional class; 6MWD: six-minute walk distance; BNP: brain natriuretic peptide. TRV: tricuspid regurgitation velocity; PASP: pulmonary artery systolic pressure, as estimated by echocardiography; TAPSE: tricuspid annular plane systolic excursion; RVFAC: right ventricular fractional area change; mPAP: mean pulmonary artery pressure; CO: cardiac output; TPR: total pulmonary resistance (mPAP/CO); and RAP: right atrial pressure.

Our second patient was a 69-year-old nonsmoker male on sildenafil. The surgical risk was high, coronary angiography showing a 90% narrowing of the mid-segment right coronary artery and an 80% narrowing of the ostium of the posterior descending artery. Therefore, in March of 2023 multimodal treatment began by stenting the coronary vessels (Figure 1), with the patient being started on riociguat. Subsequently, branches A2, A4, A5, A7, A8, A9, and A10 on the left side were submitted to BPA. Finally, right-sided PEA was uneventfully performed in December of 2023, with a total ECC time of 290 min, an aortic cross-clamp time of 40 min, and a circulatory arrest of 10 min; again, the left side was not explored. Extubation and discharge from the ICU and the hospital occurred on PODs 1, 5, and 12, respectively. At three months after surgery, the patient was in WHO functional class I and otherwise asymptomatic. 

CTEPH has encouraging survival rates when properly treated. Lifelong anticoagulation is of utmost importance.^2^ Target-specific drugs for pulmonary hypertension are aimed at vasodilating and remodeling areas of preserved flow, reducing pulmonary vascular resistance, increasing cardiac output, and slowing the progression of vasculopathy. The soluble guanylate cyclase stimulator riociguat is the first-line drug.^3^ However, mechanical treatments are the most effective and should be offered as the first choice.^4^ PEA results in long-term survival rates > 80% and mortality rates of < 5% in specialized centers.^5^ Nonetheless, only a few centers have the required technical expertise. BPA has gained momentum as an alternative treatment for inoperable disease and high surgical risk candidates. BPA targets small-caliber vessels but is not useful for central obstruction. 

A nonrandomized comparative study conducted in Japan demonstrated the safety of bilateral sequential BPA and PEA for very high-risk patients.^6^ In the aforementioned study,^6^ branches subjected to BPA were also surgically approached without interfering with the dissection plane. In a small case series, unilateral PEAs and contralateral BPA during the rewarming phase of surgery showed good results.^7^ Sequential multimodal treatment has recently been described as an alternative to upfront PEA in high surgical risk patients with mixed lesions, i.e*.*, surgically accessible on one side only.^8^ Drug treatment is commonly the treatment of first choice because of its immediate availability and because of evidence of improved prognosis in high-risk PEA patients with the use of target-specific drugs.^9^ The rationale is to approach mechanically (with BPA) the distal disease on the least affected side for improvement of symptoms, pulmonary vascular resistance, and cardiac output. Use of riociguat before BPA also reduces complications.^4^ The combination of drugs and BPA may lower the surgical risk, allowing the most diseased side to be surgically approached, with shorter aortic cross-clamp/cardioplegia and circulatory arrest times.^8^ At our center, the average circulatory arrest time is 38 min for bilateral cases, and in the cases reported here the most surgically challenging sides were not even explored. The surgical principles are the same for both approaches: adequate visualization of the dissection plane, circulatory arrest at 18-20°C, and complete clearance of all branches.^10^

Here, we observed the effect of combining optimized drug therapy and BPA. We believe that reduced pulmonary vascular resistance and increased cardiac output contributed to reducing the surgical risks. To the best of our knowledge, this is the first report of multimodal treatment of CTEPH in Brazil. This approach allows high-risk patients to undergo PEA safely. 

## References

[1] Humbert M, Kovacs G, Hoeper MM, Badagliacca R, Berger RMF, Brida M (2023). 2022 ESC/ERS Guidelines for the diagnosis and treatment of pulmonary hypertension. Eur Respir J.

[2] Delcroix M, Torbicki A, Gopalan D, Sitbon O, Klok FA, Lang I (2021). ERS statement on chronic thromboembolic pulmonary hypertension. Eur Respir J.

[3] Donaldson S, Ogunti R, Kibreab A, Mehari A (2020). Riociguat in the treatment of chronic thromboembolic pulmonary hypertension an evidence-based review of its place in therapy. Core Evid.

[4] Jaïs X, Brenot P, Bouvaist H, Jevnikar M, Canuet M, Chabanne C (2022). Balloon pulmonary angioplasty versus riociguat for the treatment of inoperable chronic thromboembolic pulmonary hypertension (RACE) a multicentre, phase 3, open-label, randomised controlled trial and ancillary follow-up study. Lancet Respir Med.

[5] Mayer E, Jenkins D, Lindner J, D'Armini A, Kloek J, Meyns B (2011). Surgical management and outcome of patients with chronic thromboembolic pulmonary hypertension results from an international prospective registry. J Thorac Cardiovasc Surg.

[6] Shimahara Y, Suzuki S, Fujiyoshi T, Honda S, Koizumi N, Yamashita J (2023). Balloon pulmonary angioplasty followed by pulmonary endarterectomy combination treatment for high-surgical-risk patients with chronic thromboembolic pulmonary hypertension. Interdiscip Cardiovasc Thorac Surg.

[7] Wiedenroth CB, Liebetrau C, Breithecker A, Guth S, Lautze HJ, Ortmann E (2016). Combined pulmonary endarterectomy and balloon pulmonary angioplasty in patients with chronic thromboembolic pulmonary hypertension. J Heart Lung Transplant.

[8] Jevnikar M, Solinas S, Brenot P, Lechartier B, Kularatne M, Montani D (2023). Sequential multimodal therapy in chronic thromboembolic pulmonary hypertension with mixed anatomical lesions a proof of concept. Eur Respir J.

[9] Castro MA, Piloto B, Dos Santos Fernandes CJC, Jardim C, S W, Oleas FG (2020). Use of medical therapies before pulmonary endarterectomy in chronic thromboembolic pulmonary hypertension patients with severe hemodynamic impairment. PLoS One.

[10] Fernandes CJCDS, Ota-Arakaki JS, Campos FTAF, Correa RA, Gazzana MB, Jardim CVP (2022). Brazilian Thoracic Society recommendations for the diagnosis and treatment of chronic thromboembolic pulmonary hypertension. J Bras Pneumol.

